# Loss of Function of the *E1*-*Like-b* Gene Associates With Early Flowering Under Long-Day Conditions in Soybean

**DOI:** 10.3389/fpls.2018.01867

**Published:** 2019-01-08

**Authors:** Jianghui Zhu, Ryoma Takeshima, Kohei Harigai, Meilan Xu, Fanjiang Kong, Baohui Liu, Akira Kanazawa, Tetsuya Yamada, Jun Abe

**Affiliations:** ^1^Research Faculty of Agriculture, Hokkaido University, Sapporo, Japan; ^2^Institute of Crop Science, National Agriculture and Food Research Organization, Tsukuba, Japan; ^3^Key Laboratory of Soybean Molecular Design Breeding, Northeast Institute of Geography and Agroecology, Chinese Academy of Sciences, Harbin, China; ^4^School of Life Sciences, Guangzhou University, Guangzhou, China

**Keywords:** soybean, *Glycine max*, flowering, *E1Lb*, photoperiodism, adaptation

## Abstract

Photoperiod response of flowering determines plant adaptation to different latitudes. Soybean, a short-day plant, has gained the ability to flower under long-day conditions during the growing season at higher latitudes, mainly through dysfunction of *phytochrome A* genes (*E3* and *E4*) and the floral repressor *E1*. In this study, we identified a novel molecular genetic basis of photoperiod insensitivity in Far-Eastern Russian soybean cultivars. By testcrossing these cultivars with a Canadian cultivar Harosoy near-isogenic line for a recessive *e3* allele, followed by association tests and fine mapping, we determined that the insensitivity was inherited as a single recessive gene located in an 842-kb interval in the pericentromeric region of chromosome 4, where *E1-Like b* (*E1Lb*), a homoeolog of *E1*, is located. Sequencing analysis detected a single-nucleotide deletion in the coding sequence of the gene in insensitive cultivars, which generated a premature stop codon. Near-isogenic lines (NILs) for the loss-of-function allele (designated *e1lb*) exhibited upregulated expression of soybean *FLOWERING LOCUS T* (*FT*) orthologs, *FT2a* and *FT5a*, and flowered earlier than those for *E1Lb* under long-day conditions in both the *e3*/*E4* and *E3*/*E4* genetic backgrounds. These NILs further lacked the inhibitory effect on flowering by far-red light–enriched long-day conditions, which is mediated by *E4*, but not that of red-light–enriched long-day conditions, which is mediated by *E3*. These findings suggest that *E1Lb* retards flowering under long-day conditions by repressing the expression of *FT2a* and *FT5a* independently of *E1*. This loss-of-function allele can be used as a new resource in breeding of photoperiod-insensitive cultivars, and may improve our understanding of the function of the *E1* family genes in the photoperiod responses of flowering in soybean.

## Introduction

Photoperiod response of flowering determines the adaptation of crops to a wide range of latitudes with different daylengths during growing seasons. Its regulatory mechanisms vary with plant species, and may rely on both evolutionally conserved and species-specific gene systems. In *Arabidopsis*, a long-day (LD) plant, *CONSTANS* (*CO*) plays a key role in regulation of photoperiodic flowering; transcriptional and post-translational regulation of *CO* results in accumulation of the CO protein in the late afternoon under LD conditions, which in turn activates *FLOWERING LOCUS T* (*FT*) florigen gene expression (reviewed by Andrés and Coupland, 2012; Song et al., 2013). Similarly, in rice, a short-day (SD) plant, a *CO* ortholog, *Heading date 1* (*Hd1*) (Yano et al., 2000), regulates the *FT* orthologs *Heading date 3a* (*Hd3a*) and *Rice FT-like 1* (*RFT1*) (Kojima et al., 2002; Tamaki et al., 2007). However, unlike in *Arabidopsis*, *Hd1* activates *Hd3a* expression under inductive SD conditions, but suppresses it under non-inductive LD conditions (Izawa et al., 2002). This functional switch, which is absent in *Arabidopsis*, is controlled by a complex of Hd1 with the monocot-specific CCT domain protein Grain number, plant height and heading date 7 (Ghd7) (Xue et al., 2008); Ghd7 represses the expression of the B-type response regulator *Early heading date 1* (*Ehd1*) (Doi et al., 2004), an activator of *Hd3a* and *RFT1* expression, by binding to its *cis*-regulatory region (Nemoto et al., 2016).

Soybean (*Glycine max*) has multiple *CO* orthologs (Fan et al., 2014; Wu et al., 2014), of which two pairs of homoeologs, *CO*-*like* (*COL*) *1a*/*COL1b* and *COL2a*/*COL2b*, fully complement the function of *CO* in *Arabidopsis* (Wu et al., 2014). *COL1a* overexpression in soybean causes late flowering, and artificial *COL1b* mutants flower significantly earlier than the wild type, indicating that both *COL1a* and *COL1b* function as floral suppressors under LD conditions, as in rice (Cao et al., 2015). However, unlike in the case of *Hd1*, the overexpression of *COL1a* does not promote flowering under inductive SD conditions, although it up-regulates major soybean *FT* orthologs, *FT2a* and *FT5a* (Kong et al., 2010; Cao et al., 2015).

Despite the conserved roles of *CO* and *COL* genes across plant species in photoperiodic flowering, there is no report that any *COL* genes are involved in the genetic variation of flowering time in soybean. Among the 11 major genes for flowering that have been reported so far (*E1*–*E9* and *J*, reviewed by Cao et al., 2017; *E10*, Samanfar et al., 2017), four maturity genes, *E1* to *E4*, are the main contributors to soybean adaptation to a wide range of latitudes (Liu et al., 2011; Jia et al., 2014; Jiang et al., 2014; Langewisch et al., 2014; Tsubokura et al., 2014; Zhai et al., 2014; Lu et al., 2015; Kurasch et al., 2017; Li et al., 2017). The floral repressor *E1* encodes a protein that contains a bipartite nuclear localization signal and a region distantly related to the B3 domain, and is a possible transcription factor that represses *FT2a* and *FT5a* expression (Xia et al., 2012). *E1* expression is up-regulated under LD conditions under the control of the phytochrome A (phyA) proteins E3 and E4 (Liu et al., 2008; Watanabe et al., 2009; Xia et al., 2012). *E2*, a soybean ortholog of *Arabidopsis GIGANTEA* (*GI*) (Watanabe et al., 2011), inhibits flowering under LD conditions through a pathway distinct from the phyA-regulated *E1* pathway (Xu et al., 2015; reviewed by Cao et al., 2017). *E1* has two homologs, *E1-like-a* (*E1La*) and *E1Lb*, encoded 10,640 kb apart from each other in the homoeologous region of chromosome 4 (Xia et al., 2012; Xu et al., 2015). Down-regulation of the *E1L* genes by virus-induced gene silencing (VIGS) in a cultivar deficient in the *E1* gene leads to early flowering and abolishes the night-break response, suggesting that the two *E1L* genes are also involved in the photoperiod responses of soybean (Xu et al., 2015).

Photoperiod insensitivity in soybean is conditioned by combinations of various alleles at *E1*, *E3*, and *E4* (Tsubokura et al., 2013, 2014; Xu et al., 2013; Zhai et al., 2015). *E3* and *E4* were originally identified as major genes for different responses of flowering to artificially induced LD conditions, where natural daylength was extended to 20 h with red light (R)-enriched cool white fluorescent lamps (fluorescent-long daylength; FLD) or far red light (FR)-enriched incandescent lamps (incandescent-long daylength; ILD) (Buzzell, 1971; Buzzell and Voldeng, 1980; Saindon et al., 1989). *e3* conditions flowering under the FLD condition (Buzzell, 1971), whereas *e4* does so under the ILD condition in the *e3* background (Saindon et al., 1989), suggesting that E3 and E4 are functionally diverged and have an epistatic relationship. On the basis of the functions of alleles at the three loci, Xu et al. (2013) classified ILD-insensitive cultivars into three genotypic groups: (group 1) the dysfunction of both *E3* and *E4*; (group 2) the dysfunction of *E1* in combination with that of either *E3* or *E4*; and (group 3) a combination of *e1-as* (hypomorphic allele), *e3*, and *E4*. Because *E4* inhibits flowering under ILD conditions (Saindon et al., 1989; Cober et al., 1996; Abe et al., 2003; Liu and Abe, 2010), the group 3 cultivars have novel genes that abolish or reduce ILD sensitivity. One such gene is an early-flowering allele at *qDTF-J*, a QTL for days to flowering in linkage group J, which encodes FT5a; early flowering is caused by its increased transcriptional activity or mRNA stability associated with an insertion in the promoter and/or deletions in the 3′ UTR (Takeshima et al., 2016). Here, we describe a novel loss-of-function allele at the *E1Lb* locus, which is most likely involved in the gain of photoperiod insensitivity in group 3 soybean cultivars. Our data suggest that *E1Lb* inhibits flowering under LD conditions, independently of *E1*, and play major roles in the control of flowering in soybean.

## Materials and Methods

### Plant Materials and Segregation Analysis

The indeterminate Far-Eastern Russian soybean cultivars Zeika (ZE), Yubileinaya (YU), and Sonata were crossed with the Canadian indeterminate cultivar Harosoy (L58-266; HA); ZE and YU were also crossed with a Harosoy near-isogenic line (NIL) for *e3* (PI547716; H-*e3*). The three Russian cultivars have the same genotype as H-*e3* at five maturity loci, *E1*, *E2*, *E3*, *E4*, and *E9* (*e1-as*/*e2*/*e3*/*E4*/*E9*), but unlike H-*e3* they flower without any marked delay under ILD conditions in comparison with natural daylength (ND) conditions (maximum daylength, 15.2 h) in Sapporo, Japan (43°07′N, 141°35′E) (Xu et al., 2013). The ILD condition was set at an experimental farm of Hokkaido University by extending the ND to 20 h by supplemental lighting from 2:00 to 7:00 and from 18:00 to 22:00 with incandescent lamps with a red-to-far-red (R:FR) quantum ratio of 0.72 (Abe et al., 2003). Seeds of F_2_ populations and parents were sown in paper pots (Paperpots No. 2, Nippon Beet Sugar Manufacturing Co., Tokyo, Japan) on 28 May 2013 for the crosses with HA and 26 May 2014 for crosses with H-*e3*. The pots were put under the ILD condition, and 12 days later seedlings were transplanted into soil. The progeny test was carried out for 48 F_2_ plants randomly selected from the H-*e3* × ZE cross and recombinant plants used in fine mapping. Seeds of these plants were sown in paper pots on late May in 2015 to 2017 (25 May, 2015; 28 May, 2016; and 26 May, 2017). After 12 days under the ILD condition, 15 seedlings per plant were transplanted into the same field. The number of days from sowing to the first flower opening (R1) (Fehr et al., 1971) of each plant was recorded.

### Association Test, Linkage Map Construction, and Fine Mapping

A total of 16 F_2_ plants from the H-*e3* × ZE cross were used to test the association of ILD sensitivity with simple sequence repeat (SSR) marker genotypes. They were selected based on the segregation pattern in their progeny, and included 8 plants fixed for ILD insensitivity and 8 plants fixed for ILD sensitivity. SSR markers were chosen from those located in genomic regions that harbored the soybean orthologs of *Arabidopsis* flowering genes (Song et al., 2004; Watanabe et al., 2012). The SSR markers significantly associated with ILD sensitivity were genotyped for a total of 306 F_2_ plants from the H-*e3* × ZE and H-*e3* × YU crosses to confirm the detected association. Plants recombinant in the targeted region were subjected to fine mapping; the genotypes for the target gene were estimated based on the segregation of flowering under the ILD condition in the progeny and were compared with the graphical genotypes constructed by using additional 11 BARCSOY SSR markers (Song et al., 2010) (Supplementary Table S1).

### Development of NILs

Four sets of NILs, each including one NIL for ILD insensitivity and another one for sensitivity, were developed from heterozygous inbred F_5_ plants derived from different F_2_ plants (#4 and #21) from the H-*e3* × ZE cross and those (#11 and #20) from the HA × ZE cross. The former two sets of NILs had the recessive *e3* allele, whereas the latter two had the dominant *E3* allele. These lines, together with parents and an ILD-insensitive NIL of HA for *e3* and *e4* (PI546043; H-*e3e4*), were cultivated in a growth chamber (25°C, 20-h daylength) with an average photon flux of 120 μmol m^-2^ s^-1^ and an R:FR ratio of 2.2 at 1 m below light sources, or in the field under the ILD condition (sowing date, May 26, 2018), as described above. For comparison, indeterminate NILs for alleles, *e1-nl* and *e1-as*, at *E1* (NIL-*E1*; *e2*/*E3*/*E4*/*E9*), which were developed from a heterozygous inbred F_5_ plant derived from a cross between the Japanese determinate cultivar Toyomusume (*e1-nl*/*e2*/*E3*/*E4*/*e9*) and HA, were included in the evaluation of flowering under the ILD condition.

### DNA Extraction and SSR Marker Analysis

Total DNA was extracted from trifoliate leaves of each of 150 H-*e3* × ZE and 156 H-*e3* × YU F_2_ plants as described by Doyle and Doyle (1990), and from each of 492 seeds from two F_2_ plants from the H-*e3* × ZE cross, as described by Xia et al. (2012). Each PCR mixture for SSR marker analysis contained 30 ng of total genomic DNA as a template, 0.2 μl of each primer (10 μM), 0.8 μl of dNTPs (2.5 mM), 0.1 μl of Taq DNA polymerase (Ampliqon), and 1 μl of 10× ammonium buffer (Ampliqon) in a total volume of 10 μl; amplification conditions were 35 cycles at 94°C for 30 s, 55°C for 30 s, and 72°C for 30 s. PCR products were separated by electrophoresis in 10.5% (w/v) polyacrylamide gels, stained with ethidium bromide, and visualized under UV light.

### Expression Analysis

A new fully expanded leaflet was sampled from each of four plants per parent and NIL at Zeitgeber time 3 in two different growing stages, the 2nd and 3rd leaf stages. The sampled leaves were bulked, immediately frozen in liquid N_2_, and stored at -80°C. Total RNA was isolated from frozen tissues with TRIzol Reagent (Thermo Fisher Scientific). DNase I (Takara) was used to remove genomic DNA. The complementary DNAs (cDNAs) were synthesized from 1 μg of total RNA by using the M-MLV reverse transcriptase system (Invitrogen) with an oligo (dT) 20 primer in a volume of 20 μL. Transcript levels of *E1*, *E1La*, *E1Lb*, *FT2a*, and *FT5a* were determined by quantitative real-time PCR. The PCR mixture (20 μL) contained 0.1 μL of the cDNA synthesis reaction mixture, 5 μL of 1.2 μM primer premix, and 10 μL SYBR Premix Ex Taq II (Takara). A CFX96 Real-Time System (Bio-Rad) was used. The PCR cycling conditions were 95°C for 3 min followed by 40 cycles of 95°C for 10 s, 59°C for 30 s, 72°C for 20 s, and 78°C for 2 s. Fluorescence was quantified before and after the incubation at 78°C to monitor the formation of primer dimers. The mRNA for β*-tubulin* was used for normalization. A reaction mixture without reverse transcriptase was also used as a control to confirm the absence of genomic DNA contamination. Amplification of a single DNA fragment was confirmed by melting curve analysis and gel electrophoresis of the PCR products. Averages and standard errors of relative expression levels were calculated from PCR results for three independently synthesized cDNAs. Primer sequences used in expression analyses are listed in Supplementary Table S1.

### Sequencing and Marker Analysis of *E1Lb*

The coding sequences of the three gene models, Glyma.04G143300, Glyma.04G143400 and Glyma.04G143500, were analyzed for H-*e3* and ZE. The coding sequences were amplified from the cDNAs by using primers listed in Supplementary Table S1. The amplified fragments were cloned into a pGEM-T Easy vector (Promega) and sequenced with a BigDye Terminator v3.1 Cycle Sequencing kit and an ABI PRISM 3100 Avant Genetic Analyzer (both from Applied Biosystems, Japan) according to the manufacturer’s instructions. A derived cleaved amplified polymorphic sequence (dCAPS) marker targeting a single-base deletion observed in ZE was developed to discriminate the functional *E1Lb* allele of H-*e3* from the loss-of-function *e1lb* allele of ZE. The 275-bp DNA fragment amplified from ZE by PCR with the forward primer 5′-GTGTAAACACTCAAAGTCCTT-3′ and the reverse primer 5′-CGTCTTCTTGATCTTCCAACG-3′ was digested with HpyCH4IV (New England Biolabs Japan) into two fragments, 254 bp and 21 bp, but the 276-bp fragment amplified from H-*e3* was resistant to HpyCH4IV digestion. The PCR products were treated with HpyCH4IV for 1 h and then separated by electrophoresis in 2.5% NuSieve 3:1 gel (Lonza), stained with ethidium bromide, and visualized under UV light.

### Survey of the Dysfunctional Allele in ILD-Insensitive Accessions

A total of 62 ILD-insensitive accessions including the three Russian cultivars were surveyed for the *E1Lb* genotype using the allele-specific DNA marker. They included 9 accessions from northern Japan, 26 from north-eastern China, 16 from Far-Eastern Russia, 8 from Ukraine, and 3 from Poland (Supplementary Table S2). The maturity genotypes at *E1* to *E4* of 50 accessions were determined previously by Xu et al. (2013), and those of the remaining 12 accessions were assayed according to Xu et al. (2013) and Tsubokura et al. (2014).

## Results

### Segregation of Flowering Time in F_2_ and F_3_ Populations

The three Russian cultivars are photoperiod insensitive (Xu et al., 2013). They flowered 45–47 days after sowing (DAS) under the ND condition of Sapporo, whereas H-*e3* and HA flowered approximately 5 and 10 days later, respectively. Under the ILD condition, the three cultivars and H-*e3* flowered 2–4 days and around 20 days later than under ND, respectively, whereas HA continued vegetative growth and did not develop any flower buds until the end of light supplementation (10 August, 76 DAS).

**FIGURE 1:**
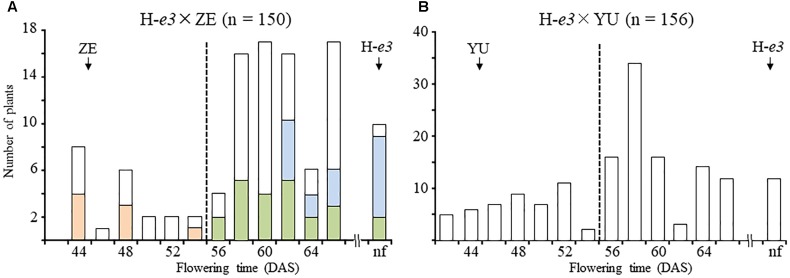
Segregation of flowering time in F_2_ populations of crosses between a Harosoy NIL for *e3* (H-*e3*) and the incandescent-long daylength (ILD)-insensitive cultivars Zeika (ZE) and Yubileinaya (YU) under far red light–enriched ILD conditions. **(A)** H-*e3* × ZE; **(B)** H-*e3* × YU. In a cross between H-*e3* and ZE, 48 F_2_ plants were selected for the progeny test; ILD-sensitivity genotypes were estimated based on the segregation in the progeny. Pink bars, homozygotes for ILD insensitivity (*e*/*e*); yellow–green bars, heterozygotes (*E*/*e*); light-blue bars, homozygotes for ILD sensitivity (*E*/*E*). Arrows indicate mean values of flowering time in parents. Dotted vertical lines indicate the threshold for classification of F_2_ plants into early-flowering ILD-insensitive and late- or non-flowering ILD-sensitive. nf, no flower buds by the end of light supplementation. DAS, days after sowing.

Flowering time under the ILD condition in F_2_ populations of the H-*e3* × ZE and H-*e3* × YU crosses varied continuously from that of ILD-insensitive parents (45 DAS for ZE and 46 DAS for YU) to the end of light supplementation; 10 out of 150 and 12 out of 156 plants had no flower buds in the H-*e3* × ZE and H-*e3* × YU F_2_ populations, respectively (Figure 1). In both populations, the distribution of flowering time tended to be bi-modal; plants which flowered at 56 DAS and later or remained vegetative segregated more than those which flowered earlier. We randomly selected 48 H-*e3* × ZE F_2_ plants and tested their progeny for flowering time segregation under the ILD condition. Based on the segregation pattern, the 48 F_2_ plants could be classified into three groups: (1) plants fixed for ILD insensitivity (all F_3_ plants tested flowered as ZE did; *e*/*e*); (2) those segregating for flowering time (*E*/*e*) and (3) those fixed for ILD sensitivity (all F_3_ plants tested showed delayed or no flowering; *E*/*E*) (Figure 1A). The number of plants was 8 in *e*/*e*, 23 in *E*/*e*, and 17 in *E*/*E*, in consistence with a monogenic 1:2:1 ratio (χ^2^ = 3.81, *df* = 2, *p* = 0.18), suggesting the involvement of a single recessive gene for ILD insensitivity. Based on the results of the progeny test, we classified 306 F_2_ plants into early-flowering ILD-insensitive plants, which flowered before 56 DAS, and late- or non-flowering ILD-sensitive plants (Figure 1). The segregation ratios of the two classes fit the expected 3:1 ratio (χ^2^ = 0.33, *df* = 1, *p* = 0.56 for H-*e3* × ZE, χ^2^ = 3.28, *df* = 1, *p* = 0.07 for H-*e3* × YU), confirming that ILD insensitivity is controlled mainly by a single recessive gene.

We also examined the segregation of flowering time under the ILD condition for the crosses between HA and the three Russian cultivars. Because HA had the *E3* allele and the three cultivars had the *e3* allele, we predicted that, in addition to the gene for ILD insensitivity segregated in the crosses with H-*e3*, the *E3* locus would also segregate in the F_2_ populations. In the three crosses, however, ILD-insensitive plants segregated at frequencies of 21.1–33.9%; the remaining plants remained vegetative until the end of light supplementation (Table 1). These segregation frequencies were thus inconsistent with those of a two-gene model, but were close to those expected from monogenic inheritance, as in the crosses with H-*e3* (Table 1).

**Table 1:** Segregation of ILD-insensitivity in F_2_ of crosses of an ILD-sensitive cultivar Harosoy with ILD-insensitive Russian cultivars.

Cross combination	Number of plants			χ^2^ value for 1:3	*P*-value
	ILD-insensitive	ILD-sensitive	Total		
Harosoy × Zeika	19	37	56	3.57	0.059
Harosoy × Yubileinaya	28	105	133	1.66	0.198
Harosoy × Sonata	19	54	73	0.06	0.803


### Association Test, Linkage Map Construction, and Fine Mapping

**FIGURE 2:**
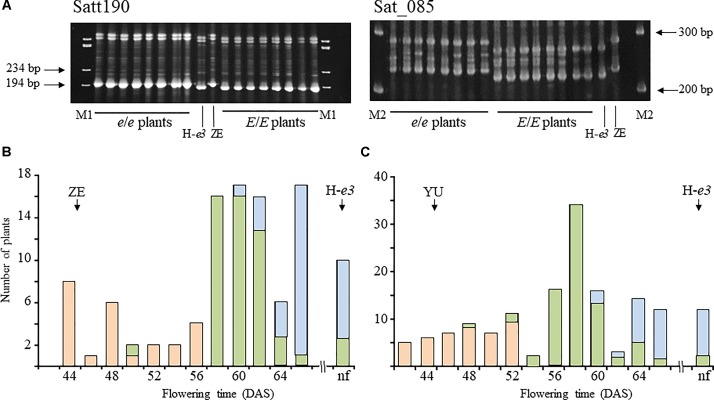
Simple sequence repeat (SSR) marker analyses in F_2_ plants from a Harosoy isoline for *e3* (H-*e3*) × Zeika (ZE) cross. **(A)** Gel electrophoresis for the analysis of Satt190 and Sat_085. Eight plants homozygous for the ILD-insensitive allele (*e*/*e*) and 8 plants homozygous for the ILD-sensitive allele (*E*/*E*) were selected on the basis of the results of the progeny test. M1, *φ*X174/HaeIII digest; M2, 100 bp DNA ladder. **(B,C)** Association between Sat_085 and flowering time in H-*e3* × ZE **(B)** and H-*e3* × YU **(C)**. F_2_ plants were classified based on the genotype at Sat_085. Pink bars, homozygotes for the allele from ILD-insensitive parents (I/I); yellow-green bars, heterozygotes (I/S); light-blue bars, homozygotes for the allele from ILD-sensitive H-*e3* (S/S). Arrows indicate mean values of flowering time in parents. DAS, days after sowing.

To determine the genomic position of the gene for ILD insensitivity from ZE, we tested the association between ILD sensitivity and SSR marker genotypes. Based on the results of the progeny test, we selected 16 F_2_ plants from the H-*e3* × ZE cross, 8 homozygous for ILD insensitivity (*e*/*e*), and 8 homozygous for ILD sensitivity (*E*/*E*). Among the SSR markers tested, Satt190 and Sat_085 in linkage group C1 (chromosome 4; Chr04) showed genotypic variation in complete accordance with the ILD sensitivity (Figure 2A). Then we determined the genotypes of the two markers in the whole F_2_ plants of H-*e3* × ZE and H-*e3* × YU populations (Figures 2B,C). The two markers were tightly linked to each other with a recombination value of 2.1, and were closely associated with ILD sensitivity. All of the plants homozygous for the allele from ILD-insensitive parents at Sat_085 (I/I) flowered before 56 DAS (H-*e3* × ZE) or 52 DAS (H-*e3* × YU), whereas those homozygous for the allele from ILD-sensitive H-*e3* (S/S) flowered at ≥60 DAS or did not flower in both crosses. Heterozygous plants (I/S) mostly flowered at ≥58 DAS (H-*e3* × ZE) or ≥54 DAS (H-*e3* × YU), which partly overlapped with the flowering date ranges of the S/S plants; only a few plants flowered as early as the I/I plants. These results strongly suggested that a gene for ILD insensitivity is located near the two SSR markers.

**FIGURE 3:**
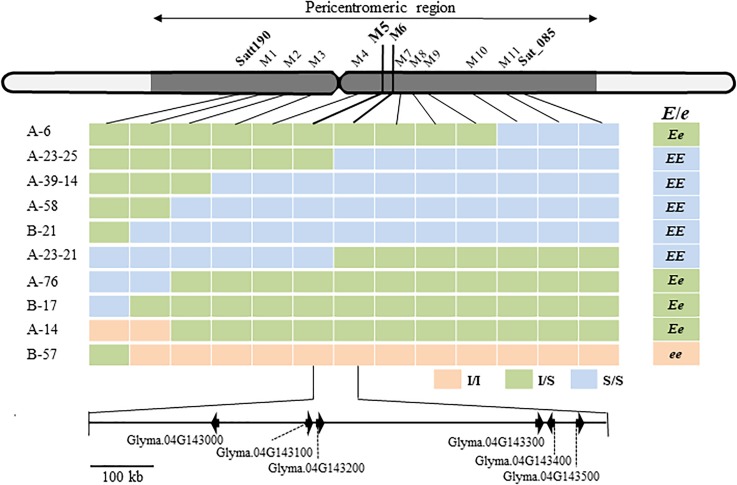
Fine mapping of a locus for ILD insensitivity (*E*/*e*) and annotated genes in the delimited region of Williams 82 chromosome 4 (*Glycine max* Wm82.a2.v1). M1 to M10, BARCSOY-SSR markers; Pink bars, homozygotes for the allele from ILD-insensitive parents (I/I); yellow–green bars, heterozygotes (I/S); light blue bars, homozygotes for the allele from ILD-sensitive H-*e3* (S/S). The *E* genotype for ILD insensitivity was estimated from the segregation patterns in the progeny. Six genes annotated in the region between M5 and M6 are shown at the bottom.

Satt190 and Sat_085 are located 17.3 Mb from each other in the pericentromeric region of Chr04 (Schmutz et al., 2010) (Phytozome v12.1/*Glycine max Wm82.a2.v1*). To delimit the genomic region of the gene for ILD insensitivity more precisely, we selected plants with recombination between the two markers (7 from 306 F_2_ plants from the H-*e3* × ZE and H-*e3* × YU crosses and 3 from 492 F_3_ plants from the H-*e3* × ZE cross) and constructed their graphical genotypes with 11 SSR markers. A comparison of the graphical genotypes with the genotype of ILD insensitivity estimated by the progeny test revealed that the gene for ILD insensitivity was located between SSR markers M5 (BARC-18g-0889) and M6 (BARC-18g-0895) (Figure 3). The physical distance between the two markers was 842 kb, and the delimited region contained only 6 annotated genes (Phytozome v12.1/*Glycine max Wm82.a2.v1*) (Figure 3 and Table 2). RNA-sequencing Atlas in Phytozome v12.1/*Glycine max Wm82.a2.v1* indicates that Glyma.04G143000, Glyma.04G143100 and Glyma.04G143200 are expressed only in flower or root tissues, whereas Glyma.04G143300, Glyma.04G143400, and Glyma.04G143500 are expressed in leaves (Severin et al., 2010). Because ZE exhibited significantly higher expressions for *FT2a* and *FT5a* in leaves in the 2nd and 3rd trifoliate leaf stages than H-*e3* under R-enriched LD condition (Supplementary Figure S1), we focused on the three genes expressed in leaves as a possible candidate of the gene for ILD insensitivity that upregulates the two *FT* genes.

**Table 2:** Genes annotated in an 842-kb genomic region in chromosome 4 delimited by fine-mapping.

No.	Gene	Annotation (Phytozome V12.1/*Glycine max Wm82.a2.v1*)	Expressed tissues
(1)	Glyma.04G143000	Diacylglycerol kinase 7	Flower
(2)	Glyma.04G143100	RNA-binding (RRM/RBD/RNP motifs) family protein	Root
(3)	Glyma.04G143200	Pectin lyase-like superfamily protein	Flower
(4)	Glyma.04G143300	AP2/B3-like transcriptional factor family protein, *E1Lb*	Leaf
(5)	Glyma.04G143400	Cytidine/deoxycytidylate deaminase family protein	Leaf, root
(6)	Glyma.04G143500	Mitochondrial substrate carrier family protein	Flower, leaf


*Data on expressed tissues are referred from Glycine max Wm82.a2.v1. (Severin et al., 2010).*

### Sequence Analysis

**FIGURE 4:**
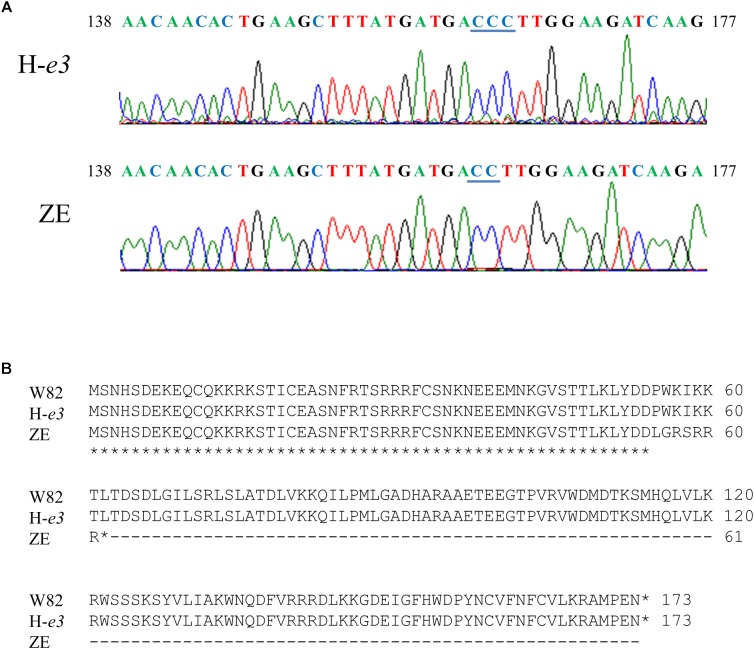
DNA and predicted amino acid sequences of Glyma.04G143300 (*E1Lb*) in Williams 82 (W82), Harosoy isoline for *e3* (H-*e3*), and Zeika (ZE). **(A)** DNA sequences of the 138th nucleotide to 177th nucleotide from the adenine of the stat codon. One of cytosines at the 162th nucleotide to 164th nucleotide underlined was deleted in ZE. **(B)** Predicted amino acid sequences.

Sequence analysis revealed that ZE and H-*e3* possessed identical sequences for Glyma.04G143400 and Glyma.04G143500, whereas one of cytosines at the 162th nucleotide to 164th nucleotide from the adenine of the start codon was deleted in the Glyma.04G143300 from ZE; this deletion generated a premature stop codon, and the Glyma.04G143300 from ZE was predicted to encode a truncated protein of 61 amino acids (Figure 4). Glyma.04G143300 is *E1Lb*, one of two homoeologs (*E1La* and *E1Lb*) of floral repressor *E1* (Xia et al., 2012). Because the down-regulation of *E1La* and *E1Lb* expressions by VIGS promotes flowering under non-inductive conditions such as LD and night break (Xu et al., 2015), we considered the loss-of-function allele of *E1Lb* (designated *e1lb* hereafter) as the most probable causal factor for the ILD-insensitivity.

**FIGURE 5:**
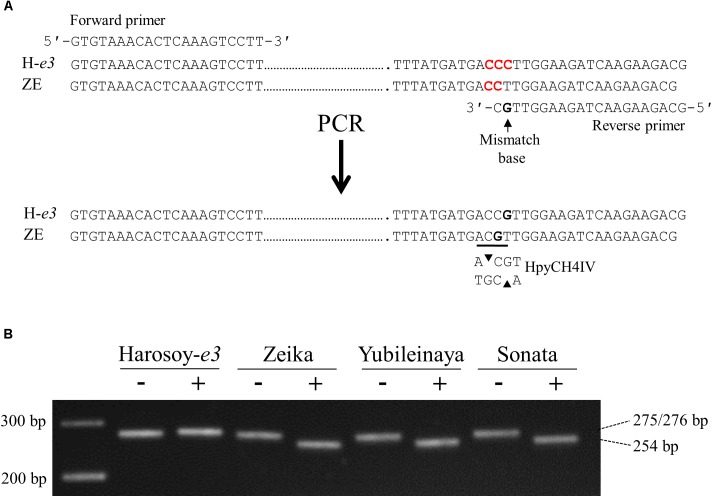
Derived cleaved amplified polymorphic sequence (dCAPS) marker analysis to detect the single-base deletion in the *e1lb* allele. **(A)** Primers designed and the generation of an HpyCH4IV restriction site. One of three cytosines marked by red was deleted in ZE. **(B)** Gel electrophoresis of PCR products without (–) or with (+) HpyCH4IV digestion. H-*e3*, Harosoy NIL for *e3*; ZE, Zeika.

We developed a dCAPS marker to discriminate *e1lb* from *E1Lb* (Figure 5). The PCR-amplified fragment of 275 bp from ZE produced a shorter fragment of 254 bp when digested with HpyCH4IV, whereas that from H-*e3* (276 bp) was not digested. The digestion of the PCR products from YU and Sonata (Russian cultivar) produced 254-bp fragments, indicating that these two cultivars had the same deletion as ZE (Figure 5B). Therefore, the segregation of ILD-insensitive plants in the crosses of these cultivars with HA and H-*e3* were most likely caused by *e1lb*.

### Comparison of Flowering Time and Gene Expression Among NILs

**FIGURE 6:**
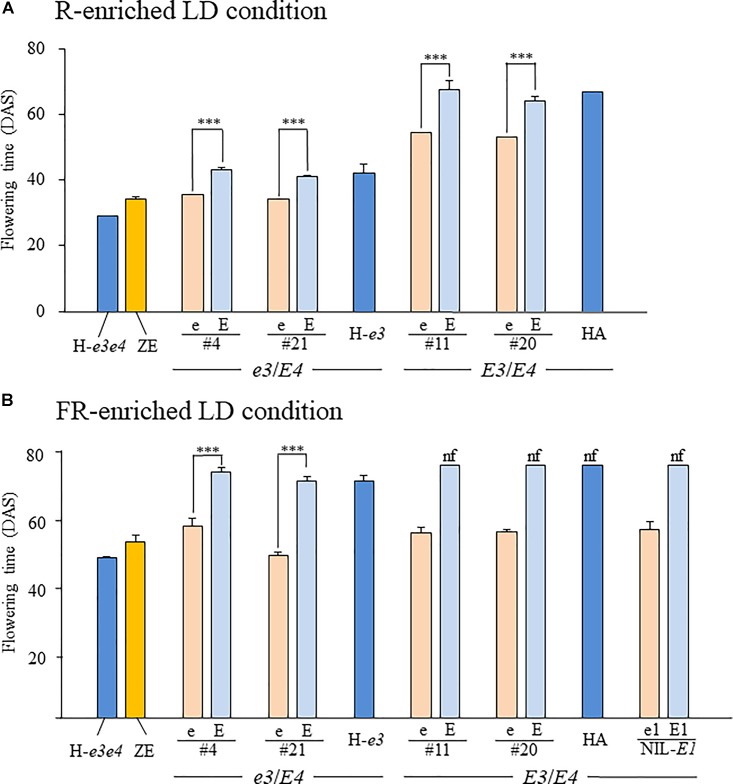
Flowering time in NILs for the *e1lb* (e) and *E1Lb* (E) alleles under R-enriched and FR-enriched LD conditions. Two sets of NILs (#4 and #21) have the *e3*/*E4* genotype, similar to H-*e3*, whereas the other two (#11 and #20) have the *E3*/*E4* genotype, similar to HA. A set of *E3*/*E4* NILs for *e1-nl* (e1) and *e1-as* (E1) at *E1* locus (NIL-*E1*) were also evaluated for the flowering under the FR-enriched LD condition. Plants were grown under 20-h **(A)** R-enriched LD or **(B)** FR-enriched ILD conditions. Data are presented as mean and standard error (*n* = 5). nf, no flower buds by the end of light supplementation, DAS, days after sowing, ^∗∗∗^*p* < 0.001.

We evaluated the allelic effects of *E1Lb* and *e1lb* on flowering under the R-enriched LD condition (daylength, 20 h) in four sets of NILs, each for *E1Lb* and *e1lb*, developed from different F_2_ plants from the H-*e3* × ZE cross (#4 and #21) and the HA × ZE cross (#11 and #20). In the two sets of the *e3*/*E4* NILs, each NIL for *e1lb* flowered at the same or almost the same time (#4, 31.7 DAS; #21, 30.3 DAS) as ZE (30.3 DAS); this was on average 6.7–7.6 days earlier than the respective NILs for *E1Lb*, which flowered at almost the same time as H-*e3* (Figure 6A). Flowering times of the *E3*/*E4* NILs were around 20 days or more later than those of the *e3*/*E4* NILs. *e1lb* also promoted flowering in the *E3*/*E4* background: each NIL for *e1lb* flowered around 10 days earlier than the respective NIL for *E1Lb* and HA. This flowering-promoting effect of *e1lb* versus *E1Lb* under the R-enriched LD condition was smaller than that of *e4* vs. *E4* and that of *e3* vs. *E3*, because H-*e3e4* and H-*e3* flowered, on average, 13 and 25 days earlier than H-*e3* and HA (*E3E4*), respectively.

We also evaluated the effect of *e1lb* vs. *E1Lb* on flowering under the FR-enriched ILD condition (Figure 6B). *e1lb* induced flowering at 58 DAS (#4) or 49 DAS (NILs #21) in the *e3*/*E4* genetic background and at 56 DAS (#11 and #20) in the *E3*/*E4* genetic background. All these NILs produced pods of up to 3 cm in length at the end of light supplementation, similar to those of ZE and H-*e3e4*. In contrast, the *e3*/*E4* NILs for *E1Lb* and H-*e3* flowered around 20 days later, and *E3*/*E4* NILs for *E1Lb* and HA continued vegetative growth and did not produce any flower buds until the end of light supplementation. Therefore, *e1lb* was sufficient to induce flowering under the ILD condition, irrespective of the *E3* genotype (Figure 6B). Interestingly, a similar flowering-promoting effect was observed in the NIL-*E1* for a loss-of-function allele *e1-nl* (e1); it initiated flowering under the ILD condition, as the *E3*/*E4* NILs for *e1lb*, whereas the NIL for *e1-as* (E1) did not (Figure 6B).

**FIGURE 7:**
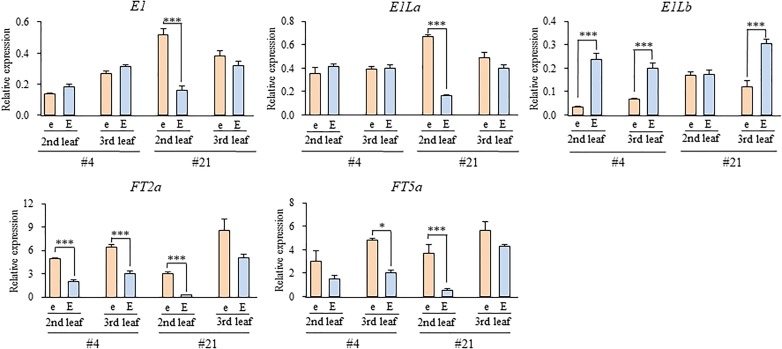
Expression levels of *E1, E1*-*Like*, and *FT* genes at the second and third leaf stages in two sets of *e3*/*E4* NILs for the *e1lb* (e) and *E1Lb* (E) alleles under R-enriched LD (20 h) conditions. Relative mRNA levels (mean and standard error, *n* = 3) are expressed as the ratios to β*-tubulin* transcript levels. ^∗^*p* < 0.05, ^∗∗∗^*p* < 0.005.

**FIGURE 8:**
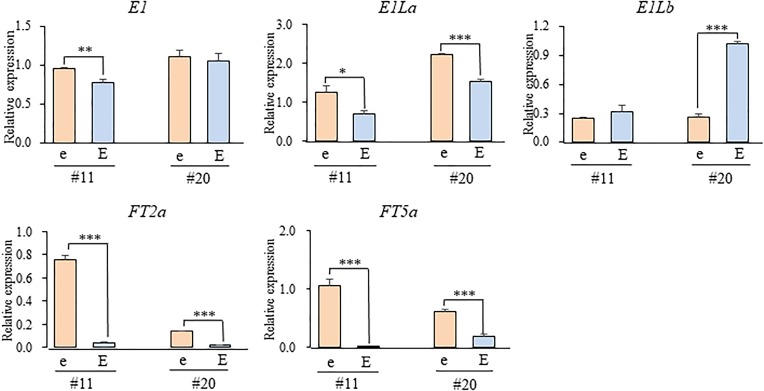
Expression levels of *E1*, *E1*-*Like*, and *FT* genes at the third leaf stage in two sets of *E3*/*E4* NILs for the *e1lb* (e) and *E1Lb* (E) alleles under R-enriched LD (20 h) conditions. Relative mRNA levels (mean and standard error, *n* = 3) are expressed as the ratios to *β-tubulin* transcript levels. ^∗^*p* < 0.05, ^∗∗^*p* < 0.01, ^∗∗∗^*p* < 0.005.

We tested the expression levels of *E1*, two *E1L* genes, and two *FT* orthologs in two different growing stages (the 2nd and 3rd leaf stages) in the *e3*/*E4* NILs grown under the R-enriched LD condition (Figure 7). The expression levels of *E1* and *E1La* were similar between the NILs for *E1lb* and *e1lb* at both stages in NILs #4 or at the 3rd stage in NILs #21; both *E1* and *E1La* were significantly up-regulated in the 2nd leaf stage in NILs (#21) for *e1lb* relative to those for *E1Lb*. On the other hand, the expression of *E1Lb* was significantly down-regulated in the NILs for *e1lb* at both stages (#4) or at the 3rd leaf stage (#21). In contrast, the expression of both *FT2a* and *FT5a* was up-regulated at both stages in the NILs for *e1lb* relative to those for *E1Lb* in both NIL sets. The similar effect of *e1lb* vs. *E1Lb* on the expression of *FT2a* and *FT5a* was also observed at the 3rd leaf stage in both sets of *E3*/*E4* NILs (#11 and #20; Figure 8). As observed in the *e3*/*E4* NILs for *e1lb*, the expression levels of *FT2a* and *FT5a* were significantly upregulated in the *E3*/*E4* NIL for *e1lb*.

### Survey of the *e1lb* Allele in ILD-Insensitive Soybean Accessions

To determine whether or not the deletion in the *E1Lb* gene is region specific, we surveyed polymorphism in the ILD-insensitive soybean accessions analyzed for the genotypes of *E1* to *E4* by using the developed dCAPS marker. In addition to the three Russian cultivars, we found that another two Russian cultivars, Salyut 216 and DYA-1, had the *e1lb* allele, whereas all the other accessions had the functional *E1Lb* allele (Supplementary Table S2). All of Russian cultivars with *e1lb* possessed the maturity genotype of *e1-as*/*e3*/*E4*. There was no cultivar which has loss-of-function alleles at both *E1* and *E1Lb* loci.

## Discussion

The soybean maturity loci, *E1* to *E4*, are major flowering loci that determine the ability of cultivars to adapt to different latitudes. Diverse allelic combinations at the *E1*, *E3*, and *E4* loci, each of which has multiple loss-of-function alleles (Tsubokura et al., 2013, 2014; Xu et al., 2013; Jiang et al., 2014; reviewed by Cao et al., 2017), have resulted in cultivars with various sensitivities to photoperiod. Photoperiod insensitivity is an adaptive trait for cultivars at high latitudes; such cultivars are classified into three genotypic groups according to the allelic functions at each of the three loci (Xu et al., 2013). Among the ILD accessions tested, the predominant group has the loss-of-function alleles of the *phyA* genes *E3* and *E4* (*e3*/*e4*), followed by a group which has the loss-of-function of the *E1* repressor for *FT2a* and *FT5a* in combination with a dominant *E3* or *E4* allele. Cultivars of the third group have a novel genetic mechanism that abolishes or reduces sensitivity to daylength, because they have the same genotype (*e1-as*/*e3*/*E4*) as an HA NIL for *e3*, which is sensitive to FR-enriched ILD conditions (Saindon et al., 1989; Cober et al., 1996; Abe et al., 2003; Liu and Abe, 2010; Xu et al., 2013). Takeshima et al. (2016) carried out QTL analysis of ILD insensitivity by a testcross of a Chinese cultivar of group 3 with the HA NIL for *e3* and demonstrated that an early-flowering allele at *qDTF-J*, which encodes the FT5a protein, up-regulates *FT5a* expression by *cis*-activation in the presence of *E4* to induce flowering under ILD conditions.

In the present study, we detected a novel loss-of-function allele that resulted from a frameshift mutation at the *E1Lb* locus in Far-Eastern Russian group 3 photoperiod-insensitive cultivars. *E1Lb* and its tandemly linked homolog, *E1La*, have high sequence similarity to *E1*, suggesting their functional similarity, although a certain degree of subfunctionalization is suggested by the presence of a number of amino acid substitutions and indels between the *E1* and *E1L* genes (Xia et al., 2012). Down-regulation by VIGS revealed that, similar to *E1*, *E1L* genes inhibit flowering under LD and night-break conditions (Xu et al., 2015), but the function of each homolog has remained undetermined. Comparison of NILs for *E1Lb* and *e1lb* in this study suggests that *e1lb* promotes flowering under both R-enriched and FR-enriched LD conditions. In particular, the effect of *e1lb* vs. *E1Lb* in the FR-enriched LD condition was similar to that of *e4* vs. *E4*, irrespective of the *E3* genotype, suggesting that *e1lb* completely cancels the inhibitory effect of FR-enriched LD on flowering modulated by *E4*. These flowering-promoting effects are most likely due to the up-regulation of *FT2a* and *FT5a*; their expression levels were not associated with the expression levels of *E1* and *E1La*. One likely explanation for this observation is that the total expression level and/or activity of *E1*, *E1La* and *E1lb* may be important for the repression of *FT2a* and *FT5a* expression. The induction of flowering by *e1lb* in the *E3*/*E4* genetic background under ILD conditions is in good accordance with monogenic segregation observed in the crosses of HA with Russian ILD-insensitive cultivars. *e1lb* also promoted flowering under R-enriched LD conditions, but its effect was small and it could not cancel flowering inhibition by *E3* as efficiently as *e3* did. The function of *E1Lb* may therefore depend on light quality of LD. Interestingly, *e1-nl* (loss-of-function allele at the *E1* locus) could also cancel the inhibitory effect of FR-enriched LD conditions on flowering, as efficiently as *e4* could. Because the effects of *e1lb* under the *e1-as* background were similar to those of *e1-nl* under the *E1Lb* background (Figure 6B), *E1* and *E1Lb* may inhibit flowering under LD conditions, independently of each other. It may be tempting in a further study to develop double recessive lines for the loss-of-function alleles at the *E1* and *E1Lb* loci not only to elucidate the interaction between the two genes and the function of another *E1* homolog, *E1La*, but also to explore the regulatory mechanisms of these *E1* family genes by E3 and E4 under different light conditions. In addition, a further study is also needed to determine why the loss-of-function allele at *E1Lb* can singly upregulate the *FT2a* and *FT5a* expression under LD condition, even though the remaining *E1* family genes are expressed normally.

*E1*, *E2*, and *E3* have large effects on flowering in a wide range of latitudes, whereas the allelic effect of *E4* is rather limited to high latitudes (Yamada et al., 2012; Tsubokura et al., 2013, 2014; Xu et al., 2013; Lu et al., 2015). Among these four soybean genes, *E1* has the most marked effect on time to flowering (McBlain et al., 1987; Upadhyay et al., 1994; Tsubokura et al., 2014). The polymorphism of *E1* (or its flanking genomic region) largely accounts for the variation in flowering time and related agronomic traits in segregating populations of different genetic backgrounds (Yamanaka et al., 2000; Wang et al., 2004; Zhang et al., 2004; Funatsuki et al., 2005; Liu et al., 2007; Zhai et al., 2015). In contrast to the *E1* gene, only a few reports have demonstrated the presence of major genes or QTLs for flowering and maturing associated with the genomic region of Chr04 harboring *E1La* and *E1Lb* (Cober et al., 2010; Cheng et al., 2011; Watanabe et al., 2017; Kong et al., 2018). Cober et al. (2010) determined that the *E8* gene, which was identified in a photoperiod-insensitive genetic background, is located in a genomic region harboring two *E1L* genes 10, 640 kb apart from each other (Xu et al., 2015), suggesting either of *E1La* and *E1Lb* as a candidate for *E8*. The QTLs for flowering and maturity were also detected in the positions of Chr04 similar to that of *E8* (Cheng et al., 2011; Watanabe et al., 2017; Kong et al., 2018). It would be interesting to determine whether the *E8* gene is *E1La* or *E1Lb* and to identify the responsible genes for these QTLs. Genotyping with an allele-specific DNA marker in this study revealed that *e1lb* is a rare and region-specific allele even in early maturing photoperiod-insensitive cultivars, suggesting that *e1lb* has neither largely contributed to the diversity of flowering behaviors nor been used widely in soybean breeding. The *e1lb* allele may therefore be useful as a new resource to broaden the genetic variability of soybean cultivars for flowering under LD conditions at high latitudes.

## Author Contributions

JZ, BL, and JA conducted the experiments. JZ, RT, and KH conducted genetic analyses, fine-mapping, and sequencing analyses. JZ and MX developed allele-specific DNA markers and analyzed the variation of genotypes in soybean accessions. JZ, MX, and TY conducted the expression analyses. JZ and JA drafted the manuscript with edits from RT, KH, MX, FK, BL, TY, and AK. All authors read and approved the final manuscript.

## Conflict of Interest Statement

The authors declare that the research was conducted in the absence of any commercial or financial relationships that could be construed as a potential conflict of interest.
