# Supplementary material for: Loss of Function of the E1-Like-b Gene Associates With Early Flowering Under Long-Day Conditions in Soybean
Source: Front Plant Sci. 2019 Jan 8;9:1867. doi: 10.3389/fpls.2018.01867 (PMC6331540; doi:10.3389/fpls.2018.01867)
Supplement: Supplementary file 3 [file Presentation_1.PPTX]

## Slide 1
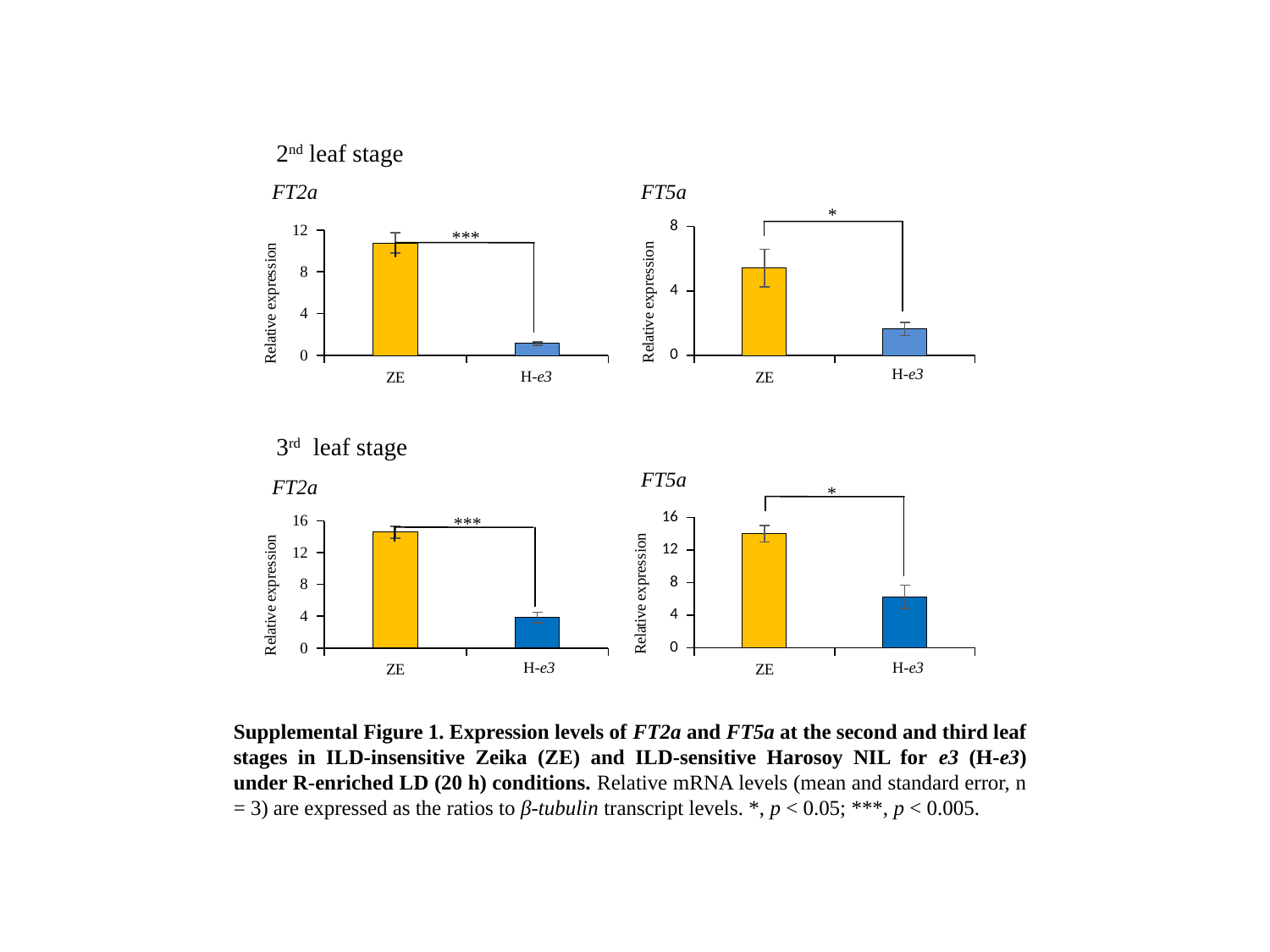

2nd leaf stage
FT5a
*
### Chart
| Category | FT5a |
|---|---|
| ZE | 5.436 |
| He3 | 1.6453333333333333 |FT5a
*
### Chart
| Category | FT5a |
|---|---|
| ZE | 13.982666666666667 |
| He3 | 6.244666666666667 |FT2a
### Chart
| Category | FT2a |
|---|---|
| ZE | 10.761333333333335 |
| He3 | 1.143 |***
FT2a
### Chart
| Category | FT2a |
|---|---|
| ZE | 14.565666666666667 |
| He3 | 3.8209999999999997 |***
3rd leaf stage
H-e3
H-e3
H-e3
H-e3
Supplemental Figure 1. Expression levels of FT2a and FT5a at the second and third leaf stages in ILD-insensitive Zeika (ZE) and ILD-sensitive Harosoy NIL for e3 (H-e3) under R-enriched LD (20 h) conditions. Relative mRNA levels (mean and standard error, n = 3) are expressed as the ratios to β-tubulin transcript levels. *, p < 0.05; ***, p < 0.005.
